# Chloride-Induced Corrosion and Mixed-Potential Control of BiHCF Electrodes in Saline Electrolytes

**DOI:** 10.3390/ijms27146389

**Published:** 2026-07-18

**Authors:** Sebastian Salazar-Avalos, Luis Cáceres, Alvaro Soliz, Pedro Pablo Zamora, Klaus Bieger, Douglas Olivares, Atul Sagade, Maritza Páez, Víctor M. Jiménez-Arévalo, Norman Toro, Felipe M. Galleguillos-Madrid

**Affiliations:** 1 Centro de Desarrollo Energético Antofagasta, Universidad de Antofagasta, Antofagasta 1240000, Chile; sebastian.salazar@uantof.cl (S.S.-A.); douglas.olivares@uantof.cl (D.O.); victor.jimenez@uantof.cl (V.M.J.-A.); 2 Departamento de Ingeniería Química y Procesos de Minerales, Universidad de Antofagasta, Antofagasta 1271155, Chile; luis.caceres@uantof.cl; 3 Departamento de Ingeniería en Metalurgia, Universidad de Atacama, Copiapó 1530000, Chile; alvaro.soliz@uda.cl; 4 Departamento de Química y Biología, Facultad de Ciencias Naturales, Universidad de Atacama, Copiapó 1530000, Chile; pedro.zamora@uda.cl (P.P.Z.); klaus.bieger@uda.cl (K.B.); 5 Solar Energy Research Center, SERC Chile, Universidad de Antofagasta, Antofagasta 1270300, Chile; 6 Departamento de Ingeniería Mecánica, Universidad de Tarapacá, Arica 1100000, Chile; asagade@academios.uta.cl; 7 Departamento de Química de los Materiales, Universidad de Santiago de Chile, Santiago 9170022, Chile; maritza.paez@usach.cl; 8 Faculty of Engineering and Architecture, Universidad Arturo Prat, Iquique 1110939, Chile; notoro@unap.cl

**Keywords:** bismuth hexacyanoferrate (BiHCF), Prussian blue analogue (PBA), mixed-potential theory, corrosion, saline electrochemistry, and brine electrolysis

## Abstract

Bismuth hexacyanoferrate (BiHCF), a Prussian blue analogue containing redox-active Fe–CN–Bi coordination motifs, was investigated as a model electrode for cathodic processes in chloride-rich saline and hypersaline electrolytes. Rather than evaluating BiHCF solely as a hydrogen evolution catalyst, this work focuses on the coupled electrochemical and interfacial processes that govern its response in NaCl solutions and natural brines from seawater, reverse osmosis (RO) reject, and high-altitude brine environments. Structural characterization by SEM–EDS, XRD and FTIR confirmed the formation of crystalline BiHCF with rod-like micrometric morphology and preserved cyanide coordination. Linear sweep voltammetry under controlled hydrodynamic conditions revealed a progressive cathodic displacement of the mixed potential with increasing NaCl concentration, together with a marked suppression of oxygen reduction kinetics at high chloride activity. Mixed-potential analysis showed that HER kinetics remain comparatively less sensitive to salinity than ORR, whereas the anodic contribution associated with BiHCF oxidation becomes strongly affected by chloride-induced surface transformation. Post-electrochemical characterization indicates the formation of a BiOCl-rich surface layer when the BiHCF is in contact with a hypersaline electrolyte during the cathodic subprocess (close to 0 mV_SHE_), which accounts for the transition from active mixed-control behaviour to a passivated interfacial regime. Density functional theory calculations suggest that elementary water activation and hydrogen-forming steps at Bi sites are intrinsically feasible, implying that the experimentally observed overpotentials originate primarily from transport, interfacial resistance and chloride-driven passivation rather than from an unfavourable molecular reaction pathway. These findings provide a mechanistic framework for understanding Bi-based Prussian blue analogue electrodes in non-purified saline electrochemical systems and highlight the dual role of chloride as both a charge-compensating electrolyte species and a passivating reactant.

## 1. Introduction

Electrochemical operation in saline and hypersaline electrolytes is fundamentally different from conventional water electrolysis in purified media. In chloride-rich environments, cathodic polarization involves not only hydrogen evolution but also oxygen reduction, chloride adsorption, local pH gradients and surface reconstruction. These coupled processes generate mixed-potential behaviour and can induce the formation of passivating oxychloride phases, making the interpretation of electrode performance challenging [1].

The Atacama Desert has diverse aqueous environments, seawater, RO rejects, and high-altitude Salar, which serve as a global natural laboratory [2,3,4]. Brine electrolysis offers a promising way to convert industrial effluents into energy, working under harsh conditions. Yet, challenges like chloride ions, chlorine gas formation, and alkaline environments near the cathode hinder progress. Success depends on developing stable, affordable, and selective electrocatalysts, avoiding costly noble metals like platinum and iridium to enable large-scale use [5].

Despite this potential, the electrochemical operation in chloride-containing media remains highly challenging due to the complex interplay between competing cathodic reactions and corrosion processes. In saline systems, the hydrogen evolution reaction (HER) and oxygen reduction reaction (ORR) coexist within overlapping potential windows, while high chloride concentrations promote surface adsorption, alter interfacial charge transfer, and induce the formation of passivating layers. These effects lead to significant deviations from ideal kinetic behaviour, often resulting in mixed-control regimes governed by both charge-transfer and mass transport limitations. Consequently, understanding the coupling between HER, ORR, and surface transformations under saline conditions is essential for evaluating electrode performance and stability.

In this context, Prussian blue analogues (PBAs) are a diverse group of coordination compounds made up of three-dimensional cyanometal networks with the general formula M_3_[M′(CN)_6_]·nH_2_O [6,7,8]. They have an open cubic structure with octahedral units linked by cyanide bridges, enabling electron and ion transport through channels. Due to their versatility, adjustable stoichiometry, and low cost, PBAs are promising for electrochemical uses like saline water splitting. Recently, they have attracted interest as sustainable materials for energy conversion. However, their behaviour under highly saline and chloride-rich environments remains insufficiently understood, particularly in terms of stability and surface transformations [9,10]. The large excess of chloride ions relative to dissolved oxygen is an intrinsic characteristic of saline and hypersaline electrolytes and, therefore, affects electrochemical interfaces irrespective of the electrode material. Under these conditions, differences in concentration, transport properties, and interfacial adsorption between Cl^−^ and O_2_ can markedly influence the accessibility of the oxygen reduction reaction (ORR).

Bismuth hexacyanoferrate (BiHCF) is a particularly interesting PBA because the Bi centres provide a highly polarizable coordination environment that may influence water activation, intermediate stabilization and interfacial charge transfer [11,12,13,14]. In addition, Bi-based materials exhibit a strong affinity for chloride-containing species. Previous studies have shown that Bi/BiOCl conversion chemistry can be exploited for chloride storage and electrochemical chloride removal, indicating that Bi-containing electrodes can interact strongly with Cl^−^ under electrochemical conditions [11]. While this affinity may be advantageous for Cl^−^ capture, it can also promote surface passivation during cathodic operation in saline media. Therefore, BiHCF offers a useful platform for studying the competition between catalytic activity and chloride-induced surface transformation [15,16].

Despite this potential, the electrochemical response of BiHCF in saline and hypersaline electrolytes has not been systematically analyzed in terms of coupled HER, ORR and chloride-driven passivation. This gap is particularly relevant for natural electrolytes such as seawater, RO reject and high-altitude brines, where chloride concentration, oxygen availability and multicomponent ionic composition can strongly affect electrode behaviour. A mechanistic understanding of these effects is required before BiHCF or related Bi-based PBAs can be considered for practical electrochemical operation in non-purified water sources.

Here, we investigate the electrochemical behaviour of BiHCF electrodes in artificial NaCl solutions and natural Cl^−^-rich electrolytes from seawater, RO reject and high-altitude brine environments, respectively. BiHCF was synthesized by controlled coprecipitation and characterized by SEM–EDS, XRD and FTIR to confirm its morphology, elemental distribution and cyanide-bridged framework. Linear sweep voltammetry under controlled hydrodynamic conditions was combined with mixed-potential analysis to deconvolute the partial contributions of HER, ORR and BiHCF oxidation. Post-electrochemical characterization was used to assess chloride-induced surface transformation, with particular attention to the formation of a BiOCl-rich during intercalation of Cl^−^ ions. Finally, density functional theory (DFT) calculations were employed to evaluate the intrinsic feasibility of water activation and H_2_-forming steps at Bi sites. By combining electrochemical kinetics, natural electrolyte testing and molecular-level modelling, this work provides a mechanistic framework for understanding the transition from active BiHCF electrodes to BiOCl-passivated interfaces in chloride-rich brines. BiHCF was selected not because Bi–Cl interactions were expected to be negligible, but precisely because the chloride-sensitive chemistry of bismuth provides an experimentally accessible model for investigating the competition between electrochemical activity and surface reconstruction in a PBA electrode.

This study therefore shifts the discussion from simple activity assessment toward interfacial mechanisms, showing that the performance of BiHCF in saline electrochemical systems is governed not only by intrinsic catalytic sites but also by chloride-driven surface reconstruction and mixed-potential coupling between cathodic and anodic processes.

## 2. Results

### 2.1. Structural, Morphological and Chemical Characterization of BiHCF

**Figure 1:**
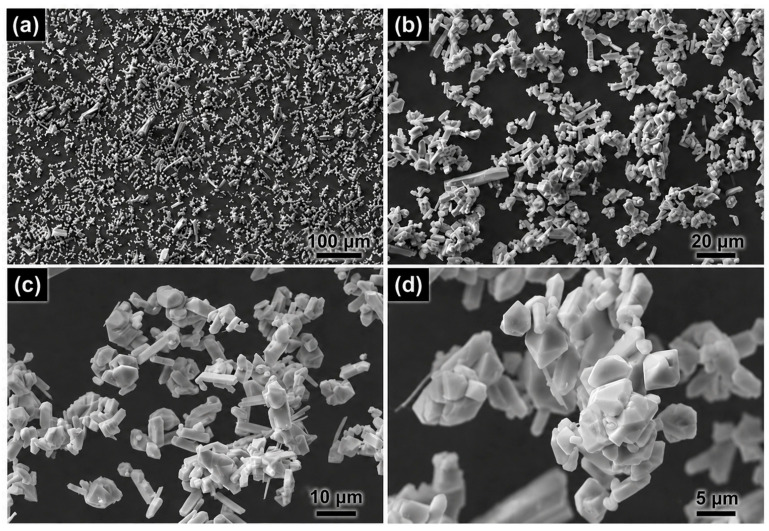
SEM images of the BiHCF powders at (**a**) 100×, (**b**) 500×, (**c**) 1000×, and (**d**) 2000×.

The morphology, elemental distribution and crystal structure of the synthesized BiHCF were first examined to establish the physicochemical features of the electrode material prior to electrochemical testing. Figure 1 presents SEM images of the BiHCF sample, providing comprehensive insights into its surface morphology. The morphological analysis of BiHCF shows that the coprecipitation route produced a bimodal particle size distribution and well-defined particle population, characterized by a combination of large irregular aggregates and smaller, regularly shaped microstructures, summarizing the main morphological features and particle size distribution parameters obtained from SEM observations and particle size analysis [17].

At 100× magnification (Figure 1a), the images revealed a heterogeneous particle distribution composed of large irregular aggregates coexisting with a population of smaller rod-like particles. However, some large, irregularly shaped particles were also observed, measuring approximately 30 μm. In comparison with SEM images at magnifications of 500× (Figure 1b) and 1000× (Figure 1c), the particles revealed a finer distribution and regular shape, with particle size on the order of 10 μm, decreasing to particle sizes close to 5 μm (Figure 1d). Additionally, the material exhibited a non-conventional morphology, showing an elongated rod with a hexagonal prism shape and rhombohedral endings, where the faces at the top and bottom of the prism had a size of approximately 5 μm (Figure 1d).

These results are in concordance with the particle size distribution, presented in Table 1, where the results reveal D10, D50 and D90 values of 4.6, 11.8 and 22.4 μm, respectively. The presence of these elongated rod-like structures is likely attributable to the specific synthesis methodology employed, which influences both the textural properties of the final material and the water content within the composite. This anisotropic morphology is consistent with diffusion-controlled nucleation and growth mechanisms typically reported for Prussian blue analogues (PBAs), where coordination-driven assembly governs particle formation [18].

Further, Xiaohan Wang et al. observed a similar morphological pattern [17] in BiHCF synthesis using sodium citrate as a chelating agent and polyvinylpyrrolidone as a capping agent, enabling the formation of highly uniform rod-like particles by facilitating consistent nucleation sites, in contrast to the BiHCF nanoplates obtained by Feng et al. [19] through amperometric methods and by adjusting the solution acidity with nitric acid.

**Figure 2:**
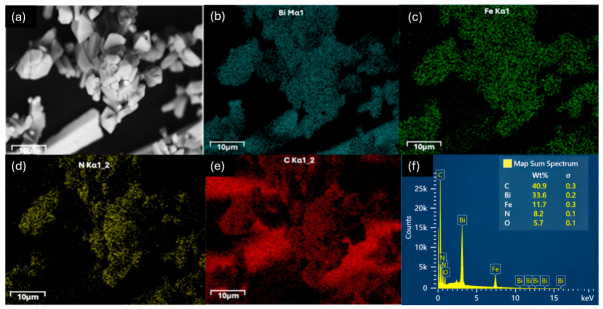
Elemental composition and spatial distribution of BiHCF powders. (**a**) SEM image and elemental mapping showing the spatial distribution of (**b**) bismuth, (**c**) iron, (**d**) nitrogen and (**e**) carbon, within the BiHCF sample. (**f**) EDS X-ray spectra for BiHCF powders (wt%).

SEM–EDS elemental mapping further supported the formation of a Bi–Fe cyanide coordination compound. As shown in Figure 2, Bi, Fe, C and N were distributed throughout the particles, indicating that the metallic centres and cyanide-related elements are spatially associated within the same solid phase. The semi-quantitative EDS analysis yielded Bi:Fe, Bi:N and Fe:N weight ratios of 4.1, 2.9 and 0.7, respectively, close to the theoretical ratios expected for the BiHCF composition considered in this work. Although EDS cannot provide definitive information on oxidation state or cyanide coordination, the simultaneous and homogeneous detection of Bi, Fe, C and N is consistent with the formation of a Bi-containing hexacyanoferrate framework rather than a simple physical mixture of metal salts or oxides.

From an electrochemical perspective, this morphology is expected to influence both charge transfer and mass transport at the electrode/electrolyte interface. The presence of elongated particles and heterogeneous size distribution may introduce non-uniform current distribution and localized diffusion gradients, which become particularly relevant under saline conditions where ionic strength and viscosity are high. Additionally, particle agglomeration can reduce the effective electrochemically active surface area, further contributing to kinetic limitations observed during HER and ORR processes.

**Figure 3:**
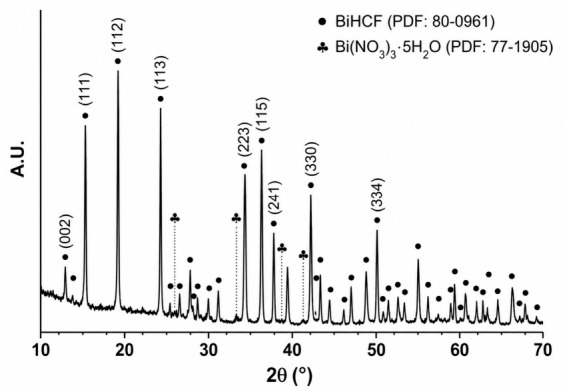
XRD constructive interference peaks of BiHCF.

The crystalline nature of the synthesized material was confirmed by X-ray diffraction (Figure 3). The diffractogram exhibited a series of well-defined reflections that can be indexed to an orthorhombic Bi–Fe cyanide hydrate phase, consistent with a Prussian blue analogue-type framework. The main reflections observed at 2θ values of 12.931°, 15.183°, 18.904°, 23.944°, 34.041°, 35.662°, 37.518°, 42.061° and 50.107° were assigned to the crystallographic planes listed in Table 2. The calculated interplanar distances ranged from 6.84 to 1.82 Å, and the estimated lattice parameter was 10.95 Å. These values are consistent with the open-framework character of PBAs and support the formation of an ordered cyanide-bridged coordination network.

The constructive interference peaks are consistent with a Bi–Fe cyanide hydrate phase related to Prussian blue analogue frameworks. For comparative purposes, an apparent lattice parameter was estimated from the main reflections, a typical structural characteristic of PBAs with the general formula M_y_[Fe(CN)_6_]·xH_2_O. Importantly, no intense reflections attributable to crystalline Bi_2_O_3_, metallic Bi or iron oxide impurities were detected in the pristine sample within the resolution of the measurement. This observation indicates that the initial material is dominated by the BiHCF phase before electrochemical exposure. This point is critical for the interpretation of the post-electrochemical results, because the appearance of chloride-containing Bi phases after polarization can then be attributed to electrochemically induced surface transformation rather than to impurities already present in the as-synthesized powder [10].

The constructive interference peaks closely align with those reported in previous studies, with only minimal shifts [10,17,19]. To determine the interplanar spacing (d), Bragg’s Law was applied, enabling the calculation of lattice spacing from the observed diffraction angles (θ). The results of these calculations are summarized in Table 2, which provides d-spacing values for the synthesized BiHCF at the different planes. The average interplanar spacing obtained from the highest intensity peaks was 3.62 ± 1.4 Å, indicating the high crystallinity and structural order of the BiHCF material, with minimal variation across the diffraction peaks. These findings further confirm that the synthesized material closely aligns with the expected Prussian blue analogue structure, exhibiting well-defined peaks that are characteristic of a highly crystalline material with negligible structural disorder. Further, the absence of additional peaks associated with secondary phases or impurities confirms that the synthesized material had no major crystalline impurities, which is essential for ensuring consistent electrochemical performance and long-term stability during operation. This high level of crystallinity can be attributed to the optimized synthesis parameters used, which facilitate the uniform formation of the cyanide-bridged coordination framework. In electrocatalysis, the uniform particle distribution and crystallinity promote homogeneous electron transport, which is a critical factor for enhancing catalytic performance and overall material efficiency [9,20,21].

Table 2 has been expanded to include the experimental 2θ values and d-spacings obtained in this work together with the corresponding published reference values used for phase comparison.

Furthermore, the lattice parameter (a) was determined based on Bragg’s Law calculations and the corresponding Miller indices, with the derived interplanar spacing values further validating the structural integrity of the BiHCF material. The calculated lattice parameter corresponds to 10.95 Å, consistent with values reported in the literature for Prussian blue analogues (PBAs) [10,17,19,22,23]. BiHCF demonstrates suitability and corrosion resistance for energy-related technologies such as saline water splitting, where both stability and efficiency are critical factors.

**Figure 4:**
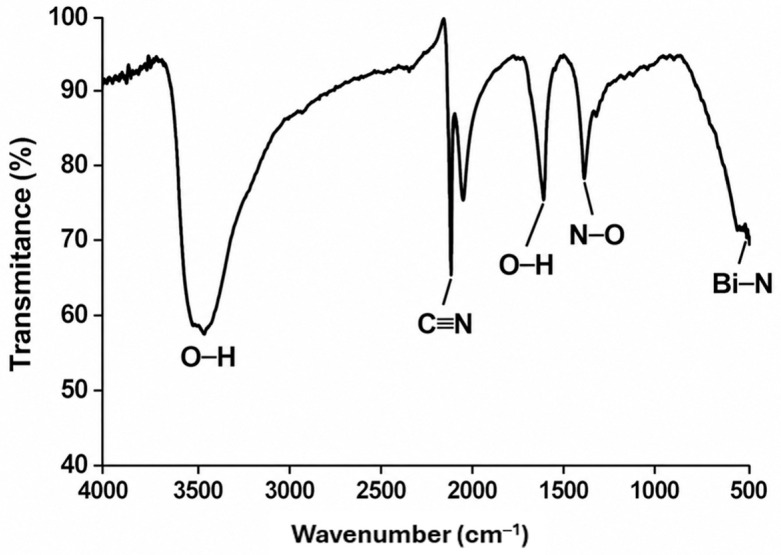
FTIR spectra of BiHCF.

The FTIR spectrum of the synthesized BiHCF material is shown in Figure 4 and provides key insights into its molecular structure and chemical composition. A prominent absorption band is observed at 2116 cm^−1^, corresponding to the stretching vibration of the C≡N triple bond.

This feature is distinctive of the cyanide ligands present in PBAs and consistent with previous literature reports [10,17,19]. Additionally, a broad absorption band centred close to 3509 cm^−1^ is attributed to O–H bonds, indicative of hydroxyl groups associated with interstitial water molecules. This observation supports the hydrated nature of the BiHCF crystal structure. Notably, a slight shift in the C≡N stretching frequency to 2119 cm^−1^ suggests electronic interaction between the cyanide ligands and transition metal ions (Fe^3+^ and Bi^3+^), reflecting subtle changes in the local ligand environment. The slight shift in the C≡N stretching frequency indicates electronic interaction between Bi^3+^ and the cyanide ligands, which may contribute to the modulation of the local electronic structure at the active sites. This effect is particularly relevant for electrochemical reactions such as the HER and ORR, where the adsorption strength of intermediates is strongly dependent on the electronic properties of the catalyst surface. Furthermore, bending vibrations at 1652 cm^−1^ and near 1375 cm^−1^ are assigned to O–H bending of water molecules linked to the crystal water in the framework and to N–O stretching vibrations of adsorbed nitrate ions within the material, respectively [17,19,24].

An additional absorption band observed at 589 cm^−1^ is associated with Bi–N stretching vibrations, providing further evidence of the coordination between bismuth ions and the cyanide framework [18,25]. The FTIR profile obtained in this study aligns closely with spectra reported for other PBAs, reinforcing the structural consistency of the synthesized BiHCF and confirming its similarity to well-characterized PBA materials [10,19]. These findings indicate that although the presence of interstitial water may influence material stability, BiHCF retains the key characteristics necessary for functional performance in applications where stability during electrochemical cycling is not a critical concern. Moreover, the observed material’s resilience in the presence of coordinated water complements the XRD results previously discussed, supporting the material’s potential in electrochemical systems.

Importantly, the presence of coordinated and interstitial water molecules within the structure may play a dual role. While they can facilitate proton transport and intermediate stabilization during electrochemical reactions, they may also reduce structural stability under prolonged operation in saline media, where ion exchange and hydration/dehydration processes are enhanced. This balance between structural integrity and electrochemical functionality becomes especially critical when the material is exposed to high-ionic-strength electrolytes.

Taken together, SEM–EDS, XRD and FTIR confirm the successful formation of crystalline BiHCF with micrometric rod-like morphology, homogeneous Bi–Fe–C–N elemental distribution and preserved cyanide coordination. These structural characteristics provide the basis for the subsequent electrochemical analysis. However, the heterogeneous particle size distribution, hydrated framework and presence of Bi centres capable of interacting with chloride species also suggest that the electrode response in saline media will likely be governed not only by intrinsic catalytic activity, but also by mass transport, surface accessibility and chloride-driven interfacial transformation.

### 2.2. Electrochemical Behaviour of BiHCF in Cl^−^-Rich Electrolytes

#### 2.2.1. Response in Artificial NaCl Electrolytes

**Figure 5:**
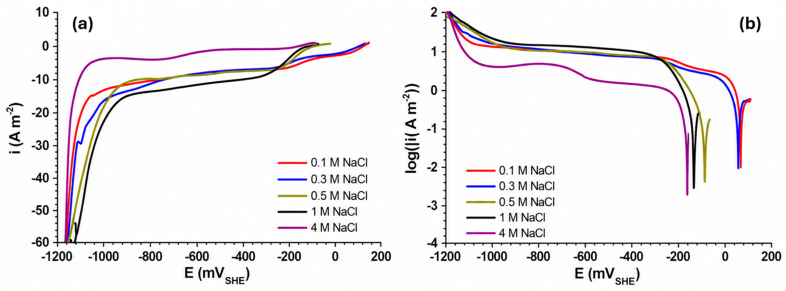
(**a**) Polarization, and (**b**) semi-logarithmic polarization curves for the BiHCF electrode in NaCl solutions. LSV analysis performed using a 2 mV s^−1^ at scan rate, aeration and WE rotation at 600 rpm.

The electrochemical response of BiHCF was first evaluated in artificial NaCl electrolytes with concentrations ranging from 0.1 to 4.0 M. This concentration window was selected to reproduce conditions from moderately saline media to hypersaline environments, allowing the effect of chloride activity and ionic strength on the coupled cathodic and anodic processes to be examined under controlled electrolyte composition. Figure 5a shows the linear sweep voltammetry curves recorded at 2 mV s^−1^ under aerated conditions and controlled electrode rotation, while Figure 5b presents the corresponding Tafel representation.

The polarization curves reveal three main electrochemical regions. At sufficiently negative potentials, the cathodic current is dominated by hydrogen evolution, although the apparent HER response may also include contributions from residual oxygen reduction depending on oxygen availability and transport. At intermediate potentials, the ORR contributes significantly to the cathodic branch, particularly in the less concentrated NaCl electrolytes. Close to the mixed potential, the total current approaches zero as the cathodic contributions are balanced by the anodic process associated with BiHCF oxidation. Therefore, the measured current cannot be assigned to a single reaction, and the electrochemical response must be interpreted using a mixed-potential framework.

A clear cathodic displacement of the mixed potential was observed as the NaCl concentration increased. The mixed potential shifted from 66 mV_SHE_ in 0.1 M NaCl to −138 mV_SHE_ in 4.0 M NaCl (Table 3). This shift indicates that the balance between cathodic and anodic partial currents is progressively modified by the electrolyte composition. In particular, the increasingly negative mixed potential suggests that oxygen reduction becomes less effective at compensating the anodic BiHCF oxidation process as the chloride concentration increases. This behaviour is consistent with the expected decrease in oxygen solubility and diffusivity in high-ionic-strength electrolytes, together with possible competitive adsorption of chloride species at the electrode/electrolyte interface.

The mixed current density also decreased markedly with increasing salinity. While values of 2.36 and 2.47 A m^−2^ were obtained in 0.1 and 0.3 M NaCl, respectively, the mixed current dropped to 0.87 A m^−2^ in 0.5 M NaCl and further decreased to 0.61 and 0.55 A m^−2^ in 1.0 and 4.0 M NaCl, respectively. This trend indicates that the global electrochemical activity of the BiHCF electrode is progressively suppressed as the chloride concentration increases. The decrease in mixed current cannot be attributed exclusively to the HER or ORR, because the total current results from the superposition of multiple partial processes. However, the simultaneous cathodic shift in mixed potential and reduction in mixed current density strongly suggest the onset of interfacial limitations at higher NaCl concentrations.

The ORR-related parameters extracted from the mixed-potential fitting provide further evidence of this behaviour. The limiting current density for oxygen reduction remained similar in 0.1–0.5 M NaCl, with values between −8.73 and −9.16 A m^−2^, and increased to −12.87 A m^−2^ in 1.0 M NaCl. However, in 4.0 M NaCl, the limiting current density decreased sharply to −1.88 A m^−2^. This sharp suppression under hypersaline conditions indicates that oxygen reduction becomes strongly hindered when chloride activity and ionic strength are sufficiently high. The non-monotonic behaviour observed between 0.1 and 1.0 M NaCl may arise from the competing effects of increasing ionic conductivity, changing oxygen transport properties and modification of the interfacial double layer. Nevertheless, the response at 4.0 M NaCl clearly reflects a transition toward a strongly transport- and/or interface-limited ORR regime.

The exchange current density associated with the ORR decreased by approximately two orders of magnitude when the NaCl concentration increased from 0.1 M to 1.0 M, changing from 2.60 × 10^−7^ to 1.47 × 10^−9^ A m^−2^. In 4.0 M NaCl, the fitted value remained in the 10^−9^ A m^−2^ range. This pronounced decrease supports the interpretation that oxygen reduction is progressively inhibited in chloride-rich electrolytes. The ORR Tafel slopes remained close to 108–114 mV dec^−1^ across the NaCl series, suggesting that the apparent reaction pathway does not change drastically in artificial NaCl media. Instead, the main effect of salinity appears to be a reduction in the number or accessibility of active sites for oxygen reduction, together with mass-transport limitations associated with lower oxygen availability in concentrated salt solutions. An additional transport asymmetry must be considered under hypersaline conditions. Chloride ions are present at molar concentrations and exhibit relatively rapid ionic transport, whereas dissolved oxygen is present at substantially lower concentration and its solubility and diffusivity decrease as salinity increases. Consequently, chloride species can continuously access and interact with the electrode/electrolyte interface while the oxygen flux required to sustain the ORR becomes progressively restricted. This transport imbalance favours increasing interfacial influence of chloride relative to oxygen at high salinity. Nevertheless, the suppression of the ORR cannot be attributed exclusively to differences in diffusion coefficients because oxygen solubility, electrolyte viscosity, water activity, competitive adsorption and chloride-induced surface reconstruction also contribute to the observed response.

In contrast, the HER-related parameters showed a comparatively weaker dependence on NaCl concentration. The HER Tafel slopes remained within a relatively narrow range, between 153 and 169 mV dec^−1^, suggesting that the apparent HER mechanism is not strongly modified by salinity within the studied concentration range. The HER exchange current density increased from 9.13 × 10^−4^ A m^−2^ in 0.1 M NaCl to 2.23 × 10^−3^ A m^−2^ in 1.0 M NaCl, before decreasing to 2.39 × 10^−4^ A m^−2^ in 4.0 M NaCl. This behaviour indicates that moderate salinity may improve charge transport and interfacial conductivity, whereas hypersaline conditions introduce additional limitations that suppress the apparent HER kinetics. Therefore, the HER on BiHCF cannot be described as simply enhanced by salinity; rather, it exhibits an optimum-like response in which conductivity benefits at intermediate NaCl concentrations are counterbalanced by interfacial blockage, reduced water activity and chloride-induced surface transformation at very high salinity. The anodic contribution associated with BiHCF oxidation was strongly affected by the electrolyte composition. Instead, they indicate that the anodic branch is governed by complex interfacial processes, likely including solid-state redox transformation, limited ion transport within the BiHCF/carbon paste matrix and the progressive formation of a passivating surface layer. The decrease in the anodic exchange current density to 0.51 A m^−2^ in 4.0 M NaCl further supports the interpretation that hypersaline media suppress the electrochemical accessibility of the BiHCF framework.

**Figure 6:**
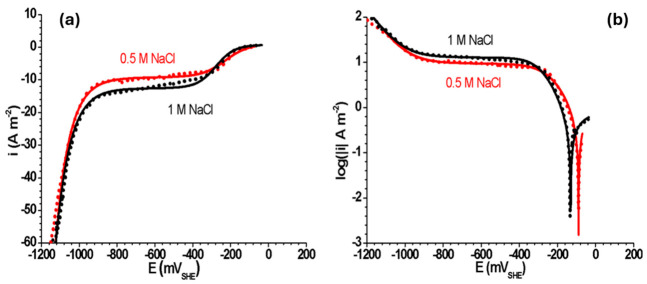
(**a**) Lineal polarization and (**b**) Tafel plots of experimental current densities (dotted curve) compared to calculated current densities (line curve) by applying the superposition model. LSV analysis performed using a 2 mV s^−1^ at scan rate, aeration and WE rotation at 600 rpm.

Figure 6 compares the experimental polarization curves with the total current density reconstructed from the superposition model. The model reproduces the main features of the cathodic and mixed-potential regions, supporting the use of mixed-potential theory to separate the contributions of HER, ORR and BiHCF oxidation. Nevertheless, small deviations between experimental and calculated currents are observed, particularly in regions where surface transformation and mass transport are expected to overlap. These deviations are reasonable considering the heterogeneous nature of the carbon paste electrode, the micrometric particle size distribution of BiHCF and the possibility of partial detachment or cracking during polarization. Therefore, the fitted parameters should be interpreted as apparent kinetic descriptors rather than intrinsic kinetic constants.

Overall, the electrochemical behaviour in artificial NaCl electrolytes demonstrates that increasing salinity progressively changes the BiHCF electrode from a more active mixed-control interface to a less active, chloride-affected regime. The ORR is the most sensitive cathodic process, being strongly suppressed at high NaCl concentrations, whereas the HER remains comparatively less affected but is also inhibited under hypersaline conditions. The anodic BiHCF contribution displays signatures of passivation rather than simple reversible oxidation. These findings indicate that chloride ions do not act solely as supporting electrolyte species; instead, they actively modify the BiHCF/electrolyte interface and prepare the conditions for the formation of BiOCl-rich passivating layers, as further examined in the post-electrochemical characterization [26,27,28].

It should be noted that the kinetic parameters obtained from the superposition model correspond to apparent values. In the present system, the measured current is affected not only by elementary charge-transfer steps, but also by oxygen transport, chloride adsorption, possible surface reconstruction, ion transport within the BiHCF/carbon paste composite and changes in the effective electrochemically accessible area. Therefore, the fitted parameters are used here to compare trends among electrolyte compositions rather than to assign intrinsic rate constants to isolated elementary reactions.

The artificial NaCl series therefore shows that BiHCF does not behave as a passive support or as a simple HER catalyst. Instead, its electrochemical response is governed by the progressive rebalancing of ORR, HER and BiHCF oxidation as chloride concentration increases. This behaviour anticipates the formation of a chloride-modified, BiOCl-rich interface under more complex natural brine conditions.

#### 2.2.2. Natural Electrolytes

After evaluating BiHCF in artificial NaCl solutions, the analysis was extended to natural chloride-rich electrolytes, including seawater, RO reject and high-altitude well brine from the Atacama region. Unlike artificial NaCl media, these electrolytes contain multicomponent ionic matrices, including chloride, sodium, sulfate, magnesium, calcium, potassium and lithium species (Table 4). Therefore, their electrochemical response cannot be interpreted solely as a function of chloride concentration. Instead, pH, conductivity, dissolved oxygen content, ionic strength, viscosity and specific ion effects must also be considered when comparing their polarization behaviour.

**Figure 7:**
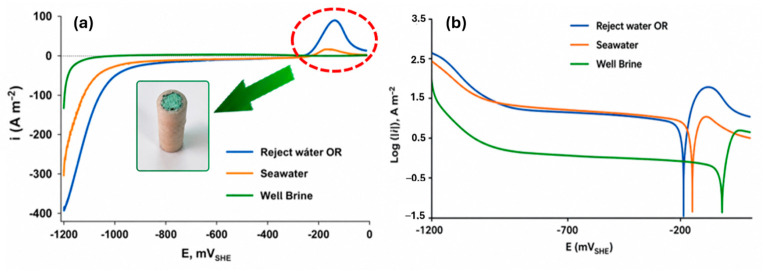
Linear polarization curves obtained in contact with OR reject water, well brine and seawater. (**a**) Lineal polarization and (**b**) Tafel plots of experimental current densities. LSV analysis performed using a 2 mV s^−1^ at scan rate, aeration and WE rotation at 600 rpm.

Figure 7 shows the polarization curves recorded for BiHCF in the three natural electrolytes under aerated and hydrodynamically controlled conditions. All curves exhibit a cathodic branch at negative potentials, followed by a mixed-potential region and an anodic contribution associated with BiHCF oxidation and/or surface transformation. Compared with the artificial NaCl series, the natural electrolytes display more complex polarization profiles, including wave-like features and current maxima that suggest overlapping contributions from ORR, HER and chloride-induced surface passivation. This behaviour is expected for natural brines, where multicomponent ionic composition can modify oxygen transport, interfacial adsorption and local pH gradients near the electrode surface.

The mixed-potential parameters obtained from the superposition model are summarized in Table 5. The high-altitude brine exhibited the lowest mixed current density, 0.37 A m^−2^, whereas RO reject and seawater showed substantially higher values of 7.44 and 9.60 A m^−2^, respectively. This difference indicates that the brine imposes stronger kinetic and/or transport limitations on the BiHCF electrode. Although the brine contains a high concentration of dissolved salts, high ionic strength does not necessarily translate into higher electrochemical activity. In hypersaline media, reduced oxygen solubility, increased viscosity, lower water activity and stronger interfacial ion pairing can suppress both oxygen reduction and charge transfer. Therefore, the low mixed current observed in the high-altitude brine is consistent with a more passivating and transport-limited environment.

The mixed potential also varied significantly among the natural electrolytes. The brine showed an E_mix_ value of −81 mV_SHE_, whereas RO reject and seawater shifted to more negative values of −218 and −188 mV_SHE_, respectively. These differences indicate that the balance between cathodic and anodic partial currents is strongly affected by electrolyte composition. The more negative mixed potentials observed in seawater and RO reject suggest a greater contribution of cathodic processes, particularly the ORR and HER, under these conditions. In contrast, the less negative mixed potential in the brine, together with its low mixed current, suggests that the electrode is rapidly driven toward a less active or partially passivated state.

The ORR-related limiting current provides further evidence of this trend. The fitted oxygen limiting current density was only −0.84 A m^−2^ in the high-altitude brine, compared with −11.84 A m^−2^ in RO reject and −12.45 A m^−2^ in seawater. This strong suppression of the ORR contribution in the brine is consistent with reduced oxygen availability and hindered oxygen transport in hypersaline media. The result also agrees qualitatively with the artificial NaCl experiments, where the ORR limiting current decreased sharply under the most concentrated NaCl condition. Therefore, both artificial and natural electrolyte results indicate that ORR is the cathodic process most sensitive to high-salinity and chloride-rich environments.

The apparent ORR Tafel parameters in natural electrolytes must, however, be interpreted with caution. The fitted ORR slopes varied widely, from −24 mV dec^−1^ in seawater to −462 and −547 mV dec^−1^ in brine and RO reject, respectively. Such dispersion indicates that the ORR branch in natural electrolytes is not governed by a simple elementary charge-transfer step. Instead, it likely reflects a combination of oxygen transport, adsorption competition, local alkalinization, surface blockage and BiHCF/BiOCl interfacial transformation. In particular, the extremely low fitted exchange current density obtained for the ORR in seawater should not be interpreted as a true intrinsic kinetic constant, but rather as a numerical descriptor arising from the fitting of strongly coupled partial processes.

The electrochemical trends observed in both artificial and natural chloride-rich electrolytes point to the progressive development of an interfacial limitation that cannot be explained solely by oxygen transport or solution resistance. In particular, the suppression of the ORR in hypersaline media, the decrease in mixed current and the appearance of wave-like polarization features suggest that the BiHCF surface undergoes chloride-induced transformation during polarization. To verify this hypothesis, post-electrochemical characterization was performed on BiHCF electrodes after exposure to chloride-containing electrolytes.

The HER-related parameters also showed strong dependence on natural electrolyte composition. The brine presented a low apparent HER exchange current density of 3.14 × 10^−6^ A m^−2^, indicating sluggish hydrogen evolution kinetics under hypersaline conditions. RO reject showed a slightly higher value of 1.96 × 10^−5^ A m^−2^, while seawater exhibited the largest apparent HER exchange current density, 1.57 × 10^−2^ A m^−2^. This trend suggests that seawater provides a more favourable interfacial environment for cathodic hydrogen evolution than the high-altitude brine. However, because the present experiments do not directly quantify H_2_ production or faradaic efficiency, this result should be described as an enhancement in apparent HER kinetics rather than definitive evidence of superior hydrogen production performance.

The parameters listed in Table 5 are apparent values obtained from mixed-potential fitting of polarization curves in chemically complex natural electrolytes. Because these media contain multiple ionic species and may induce surface transformation during polarization, the fitted parameters should be used to compare trends among electrolytes rather than as intrinsic kinetic constants for isolated HER, ORR or BiHCF oxidation reactions. Absolute values of exchange current densities are reported. i_0_, the ORR for seawater, was not physically meaningful due to overparameterization of the fitting model and is therefore not used for mechanistic interpretation.

The extremely low fitted ORR exchange current density obtained for seawater indicates that the numerical fit forced the ORR contribution toward an almost negligible kinetic exchange term while reproducing the total current through limiting-current and mixed-potential contributions. Therefore, this value should not be interpreted as a physically meaningful intrinsic exchange current density.

The anodic parameters associated with BiHCF oxidation also differ markedly among natural electrolytes. The apparent BiHCF exchange current density increased from 2.92 × 10^−6^ A m^−2^ in brine to 0.43 A m^−2^ in RO reject and 3.30 A m^−2^ in seawater. This behaviour suggests that the accessibility of the BiHCF redox framework and the extent of surface transformation are strongly controlled by the electrolyte matrix. However, as in the artificial NaCl series, the anodic branch should not be interpreted as a purely reversible Fe^2+^/Fe^3+^ process. In chloride-rich natural media, BiHCF oxidation is likely coupled to ion exchange, chloride adsorption, local hydrolysis and formation of Bi–O–Cl surface species.

The wave-like current profiles and the current feature observed around −200 mV_SHE_ support this interpretation. This electrochemical feature may be associated with the onset of surface passivation, where chloride-containing species interact with Bi sites and promote the formation of a BiOCl-rich layer. Such a layer can behave as a partially protective but ion-permeable interface: it may suppress further framework degradation while simultaneously increasing interfacial resistance and limiting access to active sites. This dual role explains why BiHCF can retain electrochemical response in natural electrolytes while showing signs of kinetic suppression and passivation.

Overall, the natural electrolyte experiments confirm that BiHCF behaviour in real chloride-rich waters is governed by more than NaCl concentration alone. The high-altitude brine imposes the strongest kinetic limitations, particularly for the ORR, whereas seawater and RO reject sustain higher apparent cathodic currents. Nevertheless, all natural media show evidence of coupled HER/ORR/BiHCF processes and chloride-induced surface transformation. These results reinforce the central conclusion obtained from artificial NaCl electrolytes: chloride-rich environments progressively convert BiHCF from an active mixed-potential electrode into a partially passivated BiHCF/BiOCl interface.

#### 2.2.3. Chloride-Induced Surface Transformation and BiOCl Passivation

**Figure 8:**
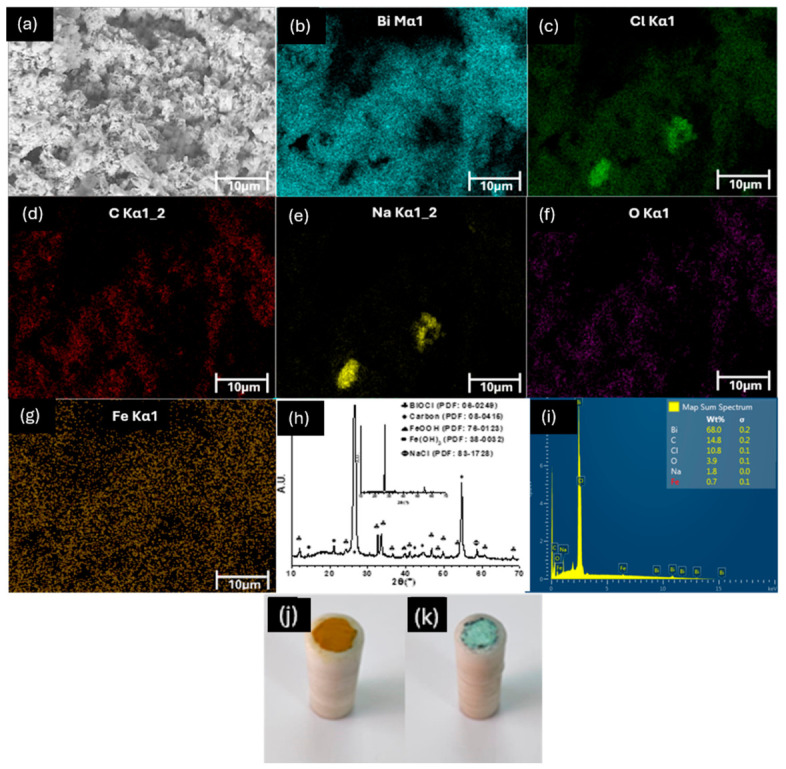
Post-electrochemical evidence of chloride-induced surface transformation of BiHCF. (**a**) SEM image of the BiHCF electrode after polarization in chloride-containing electrolyte. (**b**–**g**) EDS elemental maps showing the spatial distribution of Bi, Cl, C, Na, O and Fe after polarization. (**h**) XRD pattern of the BiHCF electrode after polarization in 0.5 M NaCl, showing diffraction features consistent with the formation of a BiOCl-rich phase. (**i**) EDS X-ray spectra. (**j**,**k**) Photographs of the BiHCF electrode before and after linear sweep voltammetry, showing macroscopic colour change associated with surface transformation.

The electrochemical trends observed in both artificial NaCl solutions and natural chloride-rich electrolytes suggest that the BiHCF electrode does not behave as a chemically inert surface during polarization. In particular, the cathodic shift in the mixed potential, the suppression of the ORR at high salinity, the decrease in mixed current density and the appearance of wave-like polarization features indicate that transport limitations are coupled with interfacial surface transformation. To evaluate this hypothesis, BiHCF electrodes were characterized after electrochemical polarization in chloride-containing electrolytes. Figure 8 shows the post-electrochemical characterization of the BiHCF electrode after linear sweep voltammetry. The samples were characterized without prior rinsing treatment after the electrochemical tests to preserve any loosely bound surface species and reaction products formed during polarization. The SEM image reveals that the electrode surface preserves a particulate morphology after polarization, but the elemental maps show a clear redistribution of chloride and oxygen over Bi-containing regions. The simultaneous presence of Bi, Cl and O after polarization is consistent with the formation of a bismuth oxychloride-rich surface layer. In contrast, the pristine BiHCF material was initially dominated by Bi, Fe, C and N signals associated with the cyanide-bridged framework, as discussed in Section 3.1. Therefore, the appearance of chloride and oxygen enrichment after polarization supports the occurrence of a chloride-induced surface transformation rather than simple retention of the original BiHCF composition.

The macroscopic colour change observed after voltammetric polarization further supports this transformation. As shown in Figure 8g,h, the electrode changes visibly after exposure to the chloride-containing electrolyte under polarization. Although colour change alone cannot identify a crystalline phase, it provides qualitative evidence of chemical modification of the electrode surface. When combined with the SEM–EDS maps and post-electrochemical XRD, this observation supports the formation of a new chloride-containing Bi phase during electrochemical operation.

The XRD pattern obtained after polarization in 0.5 M NaCl provides additional evidence for structural modification of the electrode surface. The appearance of diffraction features attributable to Bi–O–Cl species indicates that the original BiHCF framework is at least partially transformed under chloride-rich electrochemical conditions. This transformation is mechanistically plausible because Bi centres can interact strongly with chloride-containing species, particularly under local alkaline conditions generated near the cathode during oxygen reduction and hydrogen evolution. Under these conditions, the interfacial region may favour hydrolysis and chloride incorporation, leading to the formation of a BiOCl-rich layer at or near the electrode surface. The FTIR analysis performed after electrochemical polarization provides complementary information on the chemical evolution of the BiHCF coordination framework in saline and hypersaline media. Compared with the pristine BiHCF used in this study, the post-electrochemical spectrum shows modifications in the ν(C≡N) stretching region associated with the Fe–CN–Bi coordination environment, together with changes in the O–H stretching and bending regions related to coordinated water and hydroxyl-containing surface species. These spectral changes indicate that electrochemical exposure to NaCl not only results in the physical adsorption of chloride but also modifies the local chemical environment of the Bi-containing surface. The post-electrochemical SEM–EDS and XRD results, together with the FTIR analysis, provide complementary evidence of Cl^−^-induced surface reconstruction and the formation of a Bi–O–Cl-rich interphase. However, because vibrational bands associated with Bi–O- and Bi–Cl-containing environments may overlap in the low-wavenumber region, FTIR spectroscopy alone is not used here for definitive phase identification.

The formation of this layer provides a consistent explanation for the electrochemical behaviour observed throughout the study. At low and moderate NaCl concentrations, the BiHCF electrode remains sufficiently accessible to sustain mixed HER/ORR/BiHCF currents. However, as chloride concentration increases, chloride adsorption and surface reconstruction progressively reduce the accessibility of active sites and hinder oxygen reduction. At hypersaline concentration, this effect becomes dominant, as evidenced by the sharp decrease in the ORR limiting current and the lower mixed current density. Therefore, the BiOCl-rich layer acts as a passivating interphase: it can partially protect the underlying Bi-containing framework from further dissolution or degradation, but it also introduces transport limitations and increases interfacial resistance. EDS measurements were performed at a constant accelerating voltage to evaluate the depth-dependent elemental composition after voltametric testing. However, because the electron interaction volume depends strongly on the accelerating voltage, density, and local composition, the current EDS measurements provide only a semi-quantitative assessment of BiOCl formed at anodic potentials. Under hypersaline conditions, BiOCl formation may also occur at cathodic potentials.

This dual behaviour is important for interpreting the role of chloride. Chloride ions should not be regarded only as supporting electrolyte species that increase conductivity. Instead, chloride actively participates in the evolution of the electrode/electrolyte interface. At moderate concentrations, increased ionic conductivity may partially improve apparent cathodic kinetics. At high concentrations, however, chloride-driven adsorption, reduced oxygen availability, lower water activity and BiOCl-rich surface formation combine to suppress the ORR and limit the overall mixed-potential response. This explains why HER-related parameters are comparatively less sensitive to salinity than ORR-related parameters, while the anodic BiHCF contribution displays signatures of passivation rather than simple reversible oxidation.

The post-electrochemical results also clarify the meaning of the unusually high apparent anodic Tafel slopes obtained from the superposition model. These slopes should not be interpreted as elementary charge-transfer coefficients for a simple Fe^2+^/Fe^3+^ redox process. Instead, they reflect a composite process involving BiHCF oxidation, chloride adsorption, ion transport through the carbon paste/BiHCF matrix and progressive formation of a BiOCl-rich passivating interphase. Thus, the kinetic parameters extracted from the fitting describe the apparent response of a dynamically evolving electrode rather than intrinsic constants of an unchanged BiHCF surface. The BiOCl-rich layer should therefore be understood not simply as a degradation product, but as an electrochemically generated interphase that simultaneously limits charge/mass transfer and stabilizes the Bi-containing surface under chloride-rich conditions. EDS measurements were used to assess the depth dependence of the elemental composition after linear voltammetry. The observed evolution of the Cl/Bi, O/Bi, and Fe/Bi ratios is consistent with a Cl^−^ and O_2_ enriched surface region covering the BiHCF-containing electrode.

Overall, the post-electrochemical characterization supports a mechanism in which BiHCF undergoes chloride-induced surface reconstruction during polarization, generating a BiOCl-rich interphase that controls the transition from active mixed-potential behaviour to passivated response. This mechanism links the artificial NaCl and natural electrolyte results and provides a coherent explanation for the salinity-dependent suppression of the ORR, the cathodic displacement of the mixed potential and the decrease in apparent electrochemical activity under hypersaline conditions.

#### 2.2.4. Salinity-Dependent Electrochemical Regimes

Based on the artificial and natural electrolyte results, the behaviour of BiHCF in chloride-rich media can be divided into three regimes. At low salinity, represented by 0.1–0.3 M NaCl, the electrode operates in an active mixed-potential regime where the ORR contributes significantly to the cathodic current and BiHCF oxidation remains electrochemically accessible. At intermediate salinity, between 0.5 and 1.0 M NaCl, the mixed potential shifts cathodically and the HER becomes increasingly relevant, while the ORR begins to show signs of kinetic suppression. In this regime, increased ionic conductivity may still partially compensate for decreasing oxygen availability. At hypersaline concentrations, represented by 4.0 M NaCl and the well brine, BiHCF interacts with the Cl^−^ ion produced by the intercalation of the Cl^−^ ion from electrolyte within the BiHCF crystal structure, forming BiOCl. Therefore, the transition from active corrosion behaviour is controlled by the competition between conductivity enhancement, oxygen transport limitation and chloride-driven surface reconstruction. The possible involvement of Na^+^ ions in the near-surface region of the BiOCl/BiHCF interface was additionally evaluated by point EDS measurements. Since PBAs contain interstitial sites and structural vacancies capable of accommodating alkali metal ions, the retention or incorporation of sodium within the BiHCF-derived interface is chemically plausible. However, the Na^+^ detected after electrochemical exposure may also originate from residual NaCl crystallization.

### 2.3. DFT-Assisted Interpretation of Water Activation and HER-Related Steps at Bi Sites

**Figure 9:**
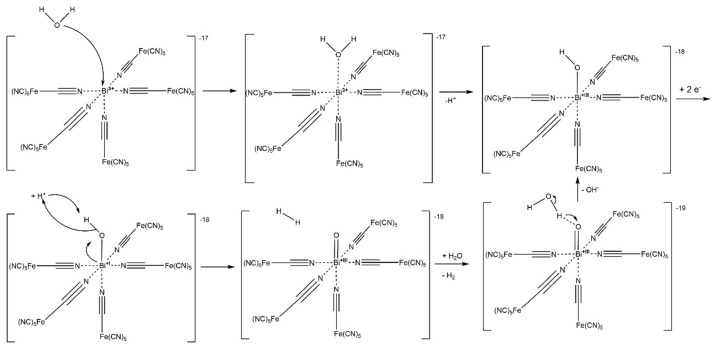
Proposed local water activation and hydrogen-forming pathway at a coordinatively accessible Bi site in the BiHCF framework. The model represents a Bi^3+^ centre coordinated by five hexacyanoferrate moieties, leaving one vacant coordination position for water adsorption. The pathway includes water coordination, deprotonation to form a Bi–OH intermediate, electron-assisted proton attack and regeneration of the Bi site.

Density functional theory calculations were performed to evaluate whether the Bi centres present in the BiHCF framework are intrinsically capable of activating water and stabilizing intermediates associated with hydrogen evolution. The purpose of this analysis is not to reproduce the full electrochemical interface, which includes the carbon paste matrix, concentrated chloride electrolytes, hydrodynamic transport and possible BiOCl-rich passivation layers, but rather to assess the local molecular feasibility of HER-related elementary steps at a representative Bi coordination environment (Figure 9). A molecular cluster containing a Bi^3+^ centre coordinated by five hexacyanoferrate moieties was selected as a simplified model of an exposed Bi site within the BiHCF framework. This configuration leaves one coordination position available for interaction with water or reaction intermediates, thereby representing a possible coordinatively accessible Bi site at the electrode/electrolyte interface. Although this model does not include long-range crystallinity, applied potential or explicit chloride-induced reconstruction, it captures the local Bi–N coordination environment relevant to the pristine BiHCF structure.

**Figure 10:**
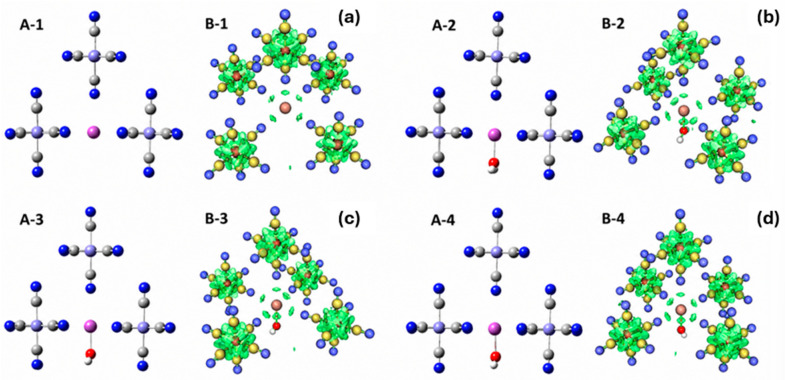
Optimized structures and reduced density gradient analysis of HER-related intermediates at the BiHCF model site. (**a**) Water-coordinated Bi site. (**b**) Bi–OH intermediate after deprotonation. (**c**) Reduced intermediate prior to proton attack. (**d**) Product state after H_2_ formation. RDG surfaces highlight attractive and repulsive non-covalent interactions involved in intermediate stabilization.

**Figure 11:**
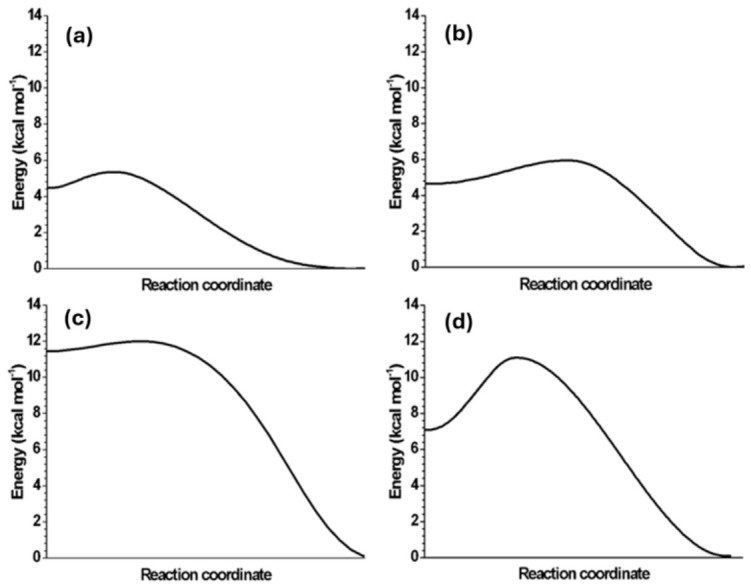
Calculated reaction coordinate profiles for water activation and HER-related steps at the BiHCF model site. (**a**) Coordination of the water molecule forming a hexacoordinated Bi^3+^–centre, (**b**) deprotonation of the coordinated water with formation of a Bi–O–H–group, (**c**) proton attack on the OH–group on the reduced intermediate, and (**d**) formation of the Bi=O–group by deprotonation. The low barriers obtained for water coordination, deprotonation and proton-assisted H_2_ formation indicate that the local Bi site does not impose a major intrinsic kinetic limitation. Energies are reported relative to the preceding intermediate.

The proposed pathway begins with coordination of a water molecule at the vacant Bi coordination site, followed by deprotonation of the coordinated water molecule to generate a Bi–OH intermediate. Subsequent electron injection facilitates proton attack on the hydroxylated intermediate, leading to molecular hydrogen formation. The catalytic cycle is completed by protonation of the remaining Bi–O species, regenerating the initial Bi coordination environment. The optimized structures of the intermediates and the corresponding reduced density gradient surfaces are shown in Figure 10, while the intrinsic reaction coordinate profiles are presented in Figure 11.

The calculations indicate that water coordination at the Bi centre is energetically accessible (Figure 11). The activation barrier for this step is approximately 6 kcal mol^−1^, and the resulting hexacoordinated intermediate is stabilized relative to the starting complex. This result suggests that exposed Bi sites can interact favourably with water molecules, an essential requirement for the HER in near-neutral saline media where proton availability is limited and water activation becomes kinetically relevant.

The subsequent deprotonation of coordinated water also proceeds with a low calculated barrier of approximately 2 kcal mol^−1^, producing a more stable Bi–OH intermediate. The stabilization of this hydroxylated species is consistent with the polarizable nature of the Bi coordination environment and with the ability of the cyanide-bridged framework to redistribute electronic density during local bond rearrangement. Reduced density gradient analysis shows attractive interactions around the Bi–O region, supporting the stabilization of the coordinated water and hydroxylated intermediates.

After the electrochemical reduction step, proton attack on the hydroxylated intermediate leads to H_2_ formation with a very low calculated barrier of approximately 1 kcal mol^−1^. The reaction products are thermodynamically favoured relative to the preceding intermediate, indicating that the local Bi site does not impose a significant intrinsic energetic penalty for hydrogen-forming elementary steps. Throughout the calculated pathway, the Bi–N bond order remains nearly preserved, decreasing only slightly from approximately 0.79 to 0.75–0.76 depending on the intermediate. This suggests that the local coordination environment can accommodate the elementary steps without severe disruption of the Bi–N framework.

These theoretical results are consistent with the experimental observation that HER-related parameters are less sensitive to salinity than ORR-related parameters. In the artificial NaCl series, the apparent HER Tafel slopes remained within a relatively narrow range, whereas ORR exchange current densities and limiting currents were strongly affected by chloride concentration. Therefore, the DFT results support the interpretation that the Bi site is intrinsically capable of participating in water activation and hydrogen-forming steps, while the experimentally observed limitations arise mainly from phenomena beyond the molecular catalytic cycle.

In particular, the high experimental overpotentials and large apparent Tafel slopes cannot be explained solely by the intrinsic energy barriers calculated for the local Bi site. Instead, they are more plausibly associated with interfacial and transport limitations, including reduced oxygen availability, changes in water activity, ohmic losses in concentrated electrolytes, heterogeneous current distribution within the carbon paste electrode and chloride-induced formation of a BiOCl-rich passivating layer. This interpretation is also consistent with the post-electrochemical characterization, which shows evidence of chloride and oxygen enrichment over Bi-containing regions after polarization.

Therefore, the DFT analysis provides a molecular-level complement to the electrochemical results. It indicates that the pristine BiHCF framework contains Bi sites that can, in principle, activate water and support HER-related elementary steps. However, under real saline and hypersaline operation, the overall electrode response is governed not by the intrinsic Bi-site reactivity alone, but by the dynamic evolution of the electrode/electrolyte interface. In this sense, the discrepancy between low calculated molecular barriers and high experimental overpotentials reinforces the central mechanistic conclusion of this work: BiHCF performance in chloride-rich media is controlled primarily by interfacial transport and chloride-driven passivation rather than by an intrinsically unfavourable HER pathway at the Bi centre.

It should be emphasized that the present DFT model does not explicitly include the applied electrode potential, explicit solvent dynamics, concentrated chloride electrolytes, carbon paste conductivity or the formation of extended BiOCl surfaces. Therefore, the calculated barriers should not be directly converted into experimental overpotentials. Instead, they are used to evaluate the local feasibility of elementary steps at Bi sites and to distinguish intrinsic molecular limitations from interfacial limitations observed experimentally.

## 3. Materials and Methods

### 3.1. Synthesis of Bismuth Hexacyanoferrate (BiHCF)

BiHCF was synthesized by the coprecipitation method [29,30], which involves the controlled dropwise addition of 25 mL of the precursor in a 30 mM aqueous solution of potassium hexacyanoferrate(II) K_4_[Fe(CN)_6_] (≥99.95%, Sigma-Aldrich, St. Louis, MO, USA) to a 75 mL 90 mM aqueous solution of bismuth nitrate pentahydrate, Bi(NO_3_)_3_·5H_2_O, also from Sigma-Aldrich, at ≥98.0%. The reaction takes place spontaneously at ambient temperature, resulting in the formation of a colloidal precipitate upon contact of the drop with the base solution. This approach enables precise regulation of the addition rate, particle size, and defect density, and was consequently employed to optimize the reaction kinetics, enhance nucleation, and thereby facilitate uniform particle formation [31,32,33]. This procedure resulted in a final solution volume of 100 mL, with a K_4_[Fe(CN)_6_] to Bi(NO_3_)_3_ molar ratio of 1:2. The resulting mixture was continually stirred with a Biologix magnetic stirrer hotplate at 600–700 rpm for 4 h at an average temperature of 60 °C to ensure complete homogenization. The precipitated solid was then collected via vacuum filtration and washed with distilled water and ethanol to remove residual impurities. The synthesized BiHCF powder was dried in a universal oven at 70 °C for 24 h, then cooled to room temperature and stored in an airtight container for later use [19,34,35]. All chemicals used were of reagent grade. The morphological and microvolume compositional characterization of the synthesized BiHCF material was performed using scanning electron microscopy coupled with EDS.

### 3.2. Surface Characterization

#### 3.2.1. Scanning Electron Microscopy (SEM-EDS)

The morphology and near-surface elemental composition of the synthesized BiHCF material were characterized by scanning electron microscopy (SEM) (Zeiss EVA MA-10, Oberkochen, Germany), coupled with energy-dispersive X-ray spectroscopy (EDS). It should be noted that, at the operating accelerating voltage of 15 kV, the SEM-EDS analysis probes a micrometric interaction volume rather than the outermost atomic surface. At a working distance of 8 mm. EDS analysis was performed in high vacuum (5 × 10^−5^ Torr). SEM images were analyzed using ImageJ software (https://imagej.net/ij/) to extract critical information on particle distribution, morphology, and grain size. Further, the particle size distribution was measured by laser scattering (Malvern Mastersize 2000, Malvern Panalytical, Westborough, MA, USA).

#### 3.2.2. X-Ray Diffraction XRD

Phases identification was carried out using X-ray diffraction (XRD) on a Shimadzu XRD-6100 system (Shimadzu XRD-600, Shimadzu Corp., Kyoto, Japan) with CuKα radiation (*λ* = 1.54056 Å). The diffraction patterns were recorded over a 2*θ* range of 10–70°, using a step size of 0.02° and a counting time of 10 s per step. Lattice parameters were determined by indexing the diffraction peaks, and the interplanar spacing was calculated using the Bragg’s Law, as given in the follow equations:

(1) λ=2·d·sinθ

(2) $$d=\frac{a}{\sqrt{h^{2}+k^{2}+l^{2}}}$$

where *λ* is the wavelength of Cu for the X-ray instrument, d is the interplanar spacing, *θ* is the Bragg angle, *a* is the lattice parameter, and h, k, and l are the Miller indices corresponding to the crystal planes.

#### 3.2.3. Fourier Transform Infrared Spectroscopy (FTIR)

Fourier Transform Infrared Spectroscopy (FTIR) analyses were performed using an Agilent Cary FTIR spectrometer equipped with an Attenuated Total Reflectance (ATR) accessory. The FTIR analyses were conducted to investigate the coordination environment of the interactions between metal ions with cyanide ligands, the presence of interstitial water molecules, and any potential structural instability inherent to the material framework. The data acquisition parameters used were transmission mode (T%); spectral range: 500–4000 cm^–1^; resolution: 4 cm^–1^; number of scans: 32; scan speed: standard for the equipment; and detector/beam-splitter: standard equipment configuration (DTGS/KBr).

### 3.3. Electrochemical Measurements

Electrochemical measurements were performed to evaluate the oxygen reduction reaction (ORR), the hydrogen evolution reaction (HER) and the BiHCF oxidation kinetics on the synthetized BiHCF material in contact with NaCl aqueous media. A conventional three-electrode electrochemical cell was employed, with a BiHCF-modified carbon paste electrode as the working electrode, a platinum wire as the counter electrode, and an Ag/AgCl reference electrode. All potential reports are referred to the standard hydrogen electrode (SHE). The working electrode was packed into a polytetrafluoroethylene (PTFE) tube (8 mm in diameter and 50 mm in length), which includes a shaft inlet for connection to a BASI/Epsilon potentiostat.

The opposite end of the tube was fitted with a bronze connector for electrical contact. The exposed surface area of the working electrode was 1.38541 × 10^−5^ m^2^. Test solutions were prepared by dissolving NaCl in distilled water to obtain solutions with concentrations of 0.1, 0.3, 0.5, 1 and 4 M NaCl (Merck S.A, Darmstadt, Germany—analytical grade). Other test solutions to consider were seawater, reject water from an osmosis plant, and well brine from salt flats located in the Atacama Desert, respectively. Electrochemical measurements for the BiHCF electrode were conducted using linear sweep voltammetry (LSV) at a scan rate of 2 mV s^−1^, with a potential window between −1200 mV_SHE_ and 0 mV_SHE_ (potential sweep in anodic direction), covering both the ORR, HER and BiHCF processes.

The working electrode was maintained at rotation rate of 600 rpm (to prevent detachment of the specimen) allowing enhancing mass transport while preventing the risk of sample detachment, which is an issue commonly observed at higher rotation speeds. The experiments were performed at 20 °C using an air conditioning system, and a continuous air bubbling system was employed to ensure the oxygen saturation of the solution. To ensure reproducibility, at least three replicates were performed for each condition. Following electrochemical polarization, the selected electrodes were removed from contact with the electrolyte without undergoing any washing procedure after voltametric testing. The samples were subsequently characterized by SEM–EDS. It should be noted that localized NaCl crystallization may contribute to the Na^+^ and Cl^−^ signals detected by EDS after the electrochemical tests. Therefore, the elemental maps are interpreted primarily as evidence of the spatial redistribution of chloride, oxygen, and bismuth rather than as definitive evidence of phase purity or the formation of a BiOCl-rich interphase.

### 3.4. Corrosion Kinetic Analysis

To elucidate the corrosion behaviour of BiHCF under cathodic operation, the electrochemical response was analyzed using mixed-potential theory and a superposition model that separates the contributions of HER, ORR, and anodic BiHCF oxidation. This approach enables quantitative assessment of how the material transitions between active, mixed-control, and passivated regimes as the chloride concentration increases. By extracting Tafel slopes, exchange current densities, and mixed potentials, the analysis reveals how the electrolyte composition governs both the catalytic performance and the intrinsic corrosive stability of the BiHCF framework.

The kinetic analysis was carried out using the superposition model and the mixed potential theory according to Equation (3), and considering mechanisms of mass diffusion, charge transfer for the cathodic ORR and HER, and the anodic reaction of BiHCF [15,26,36]. The kinetic expressions for the anodic and cathodic partial reactions were employed to deconstruct the total current density following Equations (4)–(6).

(3) $$i_{t}=i_{H_{2}}+i_{O_{2}}+i_{BiHCF}$$

(4) $$i_{H_{2}}=i_{0,H_{2}}exp\left(-\frac{2.303\cdot \eta_{H_{2}}}{b_{H_{2}}}\right)$$

(5) $$i_{O_{2}}=i_{0,O_{2}}exp\left(-\frac{2.303\cdot \eta_{O_{2}}}{b_{O_{2}}}\right)\frac{i_{l,O_{2}}}{i_{l,O_{2}}+i_{0,O_{2}}exp\left(-\frac{2.303\cdot \eta_{O_{2}}}{b_{O_{2}}}\right)}$$

(6) $$i_{BiHCF}=i_{0,BiHCF}exp\left(\frac{2.303\cdot \eta_{BiHCF}}{b_{BiHCF}}\right)$$

where $i_{t}$ is the total current density, and $i_{H_{2}}$, $i_{O_{2}}$ and $i_{BiHCF}$ are the partial current densities of the HER, ORR, and BiHCF oxidation reaction, respectively. $i_{{0,H}_{2}}$, $i_{{0,O}_{2}}$ and $i_{0,BiHCF}$ are the exchange current densities of the HER, ORR, and BiHCF oxidation reaction, respectively. $i_{{l,O}_{2}}$ is the limiting current density for the ORR, $\eta_{H_{2}}$, $\eta_{O_{2}}$ and $\eta_{BiHCF}$ are the overpotentials for the HER, ORR and BiHCF reaction, and $b_{H_{2}}$, $b_{O_{2}}\;$ and $b_{BiHCF}$ are the Tafel slopes for the HER, ORR, and BiHCF oxidation reaction, respectively.

The anodic reaction corresponds to the reversible oxidation of Fe^2+^ to Fe^3+^, which plays a central role in the electrochemical behaviour of BiHCF, as described by Equation (7).

(7) $$[Fe{\left(CN\right)}_{6}{]}^{4-}↔[{Fe\left(CN\right)}_{6}{]}^{3-}+e^{-}$$

### 3.5. Theoretical Analysis by Density Functional Theory (DFT)

Gaussian 16 was used to carry out all calculations [37]. All calculations were carried out using the ωB97X-D functional combined with the def2-TZVP basis set. The ωB97X-D functional was selected due to its range-separated formulation and inclusion of empirical dispersion corrections, which provide a reliable description of long-range interactions and electronic redistribution in coordination systems [38]. The def2-TZVP basis set was employed as a balanced triple-ζ basis suitable for both transition metals and heavy elements, ensuring a consistent description of the electronic structure across the system [39]. To characterize each step of the mechanism in this process, intrinsic reaction coordinate (IRC) curves were obtained to trace the transition states and identify activation energies along the reaction pathway. As convergence criteria for calculations, an energy variation of less than 1 × 10^−6^ Hartrees between consecutive iteration steps was applied. For gradient convergence, a maximum gradient of less than 1 × 10^−4^ Hartrees and a root mean square of less than 1 × 10^−6^ Bohr were used for the same purpose [40]. Reduced density gradient (RDG) analysis was performed to examine intra- and intermolecular interactions involved in the formation and stabilization of intermediates throughout the hydrogen production process. RDG is a mathematical function used in DFT to analyze and visualize the distribution and properties of electronic density in a molecule. It helps to identify regions with significant changes in density, such as hydrogen bonding, van der Waals interactions, or steric repulsion, that are key to understanding the stabilization of transition states and surface-bound intermediates [41]. All RDG surfaces and bond order analyses were obtained using electron densities computed at the ωB97X-D/def2-TZVP level. The gradient grid spacing was reduced to 0.5 Å. RDG visualizations and post-processing were performed using Multiwfn 3.2 [42].

## 4. Conclusions

This study demonstrates that BiHCF electrodes undergo a salinity-dependent transition from active mixed-potential behaviour to chloride-induced passivation in saline and hypersaline electrolytes. SEM–EDS, XRD and FTIR confirmed the formation of crystalline BiHCF with preserved cyanide coordination, while electrochemical measurements in artificial NaCl solutions and natural brines revealed that the electrode response is governed by the coupled contributions of HER, ORR and BiHCF corrosion. Increasing chloride concentration shifted the mixed potential toward more negative values and strongly suppressed the ORR, whereas HER-related parameters were comparatively less sensitive to salinity but became inhibited under hypersaline conditions. Natural electrolytes showed additional complexity due to their multicomponent ionic composition, confirming that chloride concentration alone is insufficient to predict electrode behaviour.

Post-electrochemical characterization provided evidence consistent with the formation of a BiOCl-rich surface layer, which explains the transition from active to partially passivated behaviour. This layer acts as an electrochemically generated interphase that can limit charge and mass transfer while modifying the stability of the Bi-containing surface. DFT calculations indicated that water activation and hydrogen-forming elementary steps at Bi sites are intrinsically feasible, suggesting that the experimentally observed overpotentials arise mainly from interfacial transport limitations and chloride-driven passivation rather than from an unfavourable molecular reaction pathway. Overall, BiHCF is best described as a dynamic chloride-responsive Prussian blue analogue electrode, and the present work provides a mechanistic basis for future design of Bi-based materials for electrochemical operation in non-purified saline waters.

These results emphasize that future development of PBA-based electrodes for saline electrochemical systems as the Atacama Desert must consider not only intrinsic catalytic activity, but also chloride-driven interfacial reconstruction, oxygen transport limitations and the dynamic formation of passivating oxychloride phases.

## Figures and Tables

**Table 1:** Morphological features and particle size distribution of BiHCF.

Parameter	Value
D10	4.6 μm
D50	11.8 μm
D90	22.4 μm
Main morphology	Rod-like/faceted particles
Secondary morphology	Irregular aggregates
Approximate aggregate size	~30 μm

**Table 2:** The diffraction peaks and Miller indices, along with the calculated interplanar distance (d).

2 θ (°)	Miller Index	d (Å)
12.931	(002)	6.84
15.183	(001)	5.83
18.904	(112)	4.69
23.944	(113)	3.71
34.041	(223)	2.63
35.662	(115)	2.52
37.518	(241)	2.40
42.061	(330)	2.15
50.107	(334)	1.82

**Table 3:** Apparent mixed-potential and kinetic parameters obtained from superposition fitting of BiHCF polarization curves in artificial NaCl electrolytes. Absolute values are reported for Tafel slopes and exchange current densities to facilitate comparison.

Parameter	0.1 M NaCl	0.3 M NaCl	0.5 M NaCl	1 M NaCl	4 M NaCl
i_mix_ (A m^−2^)	2.36	2.47	0.87	0.61	0.55
E_mix_ (mV_SHE_)	66	55	−89	−134	−138
i_L,O2_ (A m^−2^)	−8.78	−8.73	−9.16	−12.87	−1.88
b_O2_ (mV dec^−1^)	−109	−108	−113	−113	−114
i_0,O2_ (A m^−2^)	−2.60 × 10^−7^	−1.74 × 10^−7^	−5.62 × 10^−9^	−1.47 × 10^−9^	−2.14 × 10^−9^
b_H2_ (mV dec^−1^)	−166	−166	−169	−169	−153
i_0,H2_ (A m^−2^)	−9.13 × 10^−4^	−1.19 × 10^−3^	−2.04 × 10^−3^	−2.23 × 10^−3^	−2.39 × 10^−4^
i_0,BiHCF_ (A m^−2^)	1.10	2.20	0.83	4.41	0.51

**Table 4:** Chemical composition of natural electrolytes used in polarization curve analysis.

Chemical Element	Reject Water OR (mg L^−1^)	Seawater (mg L^−1^)	Well Brine (mg L^−1^)
Chloride (Cl^−^)	35.98	18,980	19,839
Sodium (Na^+^)	25.15	10,556	7933
Sulfate (SO_4_^2−^)	5.35	2649	1389
Magnesium (Mg^2+^)	2.85	1262	1746
Calcium (Ca^2+^)	0.86	400	49
Potassium (K^+^)	2.45	380	324
Lithium (Li^+^)	0.27	0.2	258

**Table 5:** Kinetics and mixed parameters of BiHCF electrodes in various natural solutions for HER, ORR, and BiHCF oxidation.

NaCl, mol/L	Well Brine	RO Rejected	Seawater
i_mix_ (A m^−2^)	0.37	7.44	9.6
E_mix_ (mV_SHE_)	−81	−218	−188
i_L,O2_ (A m^−2^)	−0.84	−11.84	−12.45
b_O2_ (mV dec^−1^)	−462	−547	−24
i_0,O2_ (A m^−2^)	1 × 10^−2^	2.3 × 10^−1^	−1.14 × 10^−41^
b_H2_ (mV dec^−1^)	−115	−105	−201
i_0,H2_ (A m^−2^)	−3.14 × 10^−6^	−1.96 × 10^−5^	−1.57 × 10^−2^
i_0,BiHCF_ (A m^−2^)	2.92 × 10^−6^	4.30 × 10^−1^	3.30

## Data Availability

The processed data are available from the corresponding author upon request.
